# Does oxygen limitation set thermal limits in aquatic ectotherms?

**DOI:** 10.1371/journal.pbio.3003441

**Published:** 2025-11-05

**Authors:** Diana Madeira

**Affiliations:** ECOMARE—Laboratory for Innovation and Sustainability of Marine Biological Resources, CESAM—Centre for Environmental and Marine Studies, Department of Biology, University of Aveiro, Gafanha da Nazaré, Portugal

## Abstract

Oxygen limitation is thought to represent a key mechanism underlying thermal tolerance. This primer discusses how a recent PLOS Biology article challenges this idea, by showing minimal protective effects of oxygen supersaturation in aquatic ectotherms exposed to heat stress.

## Teaser

Thermal tolerance determines winners and losers in a warming world. Oxygen limitation has been the primary hypothesis for the mechanistic basis of thermal tolerance. A new study in PLOS Biology suggests otherwise, reigniting debate on what truly sets thermal limits.

**Fig 1:**
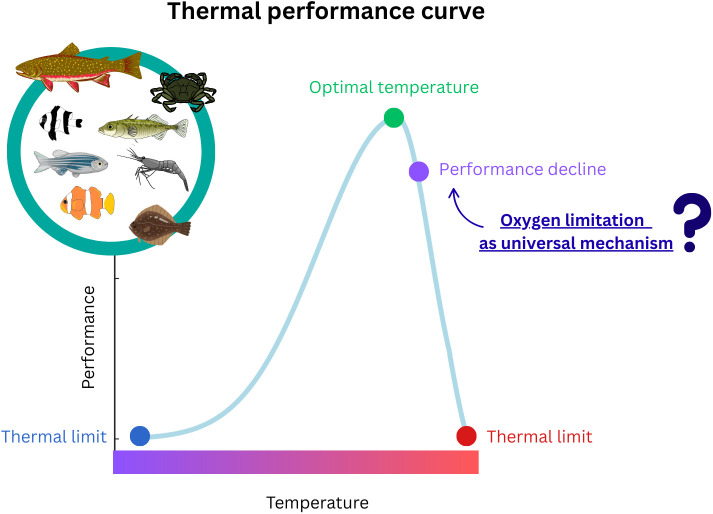
The effects of temperature on organismal performance are depicted using thermal performance curves, where performance is greatest at the optimal temperature and starts to decrease toward cooler or warmer temperatures, reflecting the thermal window (tolerable range of temperatures) of the species. Briefly, the Oxygen and Capacity Limited Thermal Tolerance hypothesis proposes that oxygen limitation explains the performance decline at high temperatures and sets the first boundary for thermal limits across ectotherms. This is due to a mismatch between the oxygen demand of the organism and the capacity of the cardiorespiratory system to supply oxygen to tissues when the organism is under warming. This concept is central to predict species responses to warming. As oxygen supersaturation in water has been shown to alleviate oxygen supply limitations by increasing maximum rates of oxygen transport in blood, Raby and colleagues (2025) tested the effects of oxygen supersaturation on thermal tolerance across 14 aquatic species. The authors found that it had negligible effects on upper thermal limits, challenging the oxygen limitation hypothesis as a universal mechanism underpinning thermal tolerance of aquatic ectotherms. The authors highlight that oxygen supersaturation in water, a naturally occurring phenomenon in shallow waters, may not protect aquatic species from the effects of extreme heat.

Environmental temperature sets the pace of life for ectotherms, which lack the ability to physiologically regulate body temperature. Thus, thermal regimes govern their molecular kinetics and reaction rates, affecting their physiology, behavior, and fitness. Ultimately, temperature effects on individual performance scale up to shape species biogeography, communities, and ecosystem dynamics. As the planet heats, whether slowly due to global warming or in sudden extreme events such as heatwaves, organisms are faced with four possible outcomes: acclimate, adapt, move, or perish. Rapid climate change has sparked interest in thermal tolerance research to identify which species are most at risk, defining winners and losers in a changing world. Over two decades ago, Pörtner 2001 [1] built on the work of Fry (1947) [2] to propose that upper thermal tolerance was, at a first instance, determined by oxygen limitation, a theory named “Oxygen and Capacity Limited Thermal Tolerance” (Fig 1). This theory provided a unifying mechanistic principle for understanding the cause–effect relationship between temperature and organismal performance, helping to predict climate change impacts on aquatic ecosystems [3]. It posits that warming leads to a mismatch between the oxygen demand of the organism and the capacity of the cardiorespiratory system to supply oxygen to tissues, setting the first boundary for whole-organism thermal tolerance. The authors argued that warming enhances oxygen limitation not only by reducing oxygen solubility in water but also by increasing organisms’ metabolic demand, which can push the cardiorespiratory system to its physiological limits at high temperature. While significant advancements were brought to the field by this theory, there has been controversy on how unifying it really is and how much empirical evidence supports it [4,5].

Research shows that oxygen supply limitations can be alleviated through oxygen supersaturation in water, as it elevates arterial oxygen partial pressure in fish [6]. In a new study in this issue, Raby and colleagues (2025) [7] tested the effects of environmental hyperoxia versus normoxia on upper thermal tolerance across a range of aquatic animals. Their study encompassed 14 species, including fish and decapod crustaceans from freshwater and marine systems, across temperate and tropical regions, in experiments with an unprecedented level of replication. The underlying hypothesis is that if oxygen limitation sets upper thermal tolerance, then oxygen supersaturation should increase thermal tolerance limits. This could be ecologically relevant, as oxygen supersaturation can naturally occur in shallow water environments, and has been proposed to buffer species from the effects of extreme heat, such as heatwaves [8]. Taking these 14 species, Raby and colleagues (2025) estimated their thermal tolerance based on a widely used metric, the Critical Thermal Maximum (CTmax) [9], which represents the temperature at which the animal loses equilibrium. They also ran fast (traditional) and slow (ecologically relevant) warming rates to test whether the rate of temperature increase played any role in how oxygen saturation affects thermal tolerance. A total of 1,451 animals were tested in experiments (7–10 *per* trial, multiple trials *per* species *per* treatment), controlling for body size.

Based on these experiments, Raby and colleagues showed that oxygen supersaturation has minor effects on thermal tolerance across the diverse set of species tested. No effect was found in 10 out of the 14 tested species, with the other 4 showing inconsistent results across replicate trials. In all cases, effect sizes were consistently small, leading to the conclusion that most water-breathers will not be protected from extreme heat by oxygen supersaturation, challenging previous predictions [8]. This aligns with other studies suggesting that oxygen limitation is not universal across species but possibly restricted to particular taxa [4,10]. However, debate persists around the use of CTmax to test the oxygen limitation hypothesis. While Pörtner and colleagues (2017) [3] have argued it is too insensitive because functional constraints on performance due to oxygen limitation occur well before this critical point, others consider it an appropriate metric [11]. An open question is therefore which approaches or metrics provide the most robust test of the oxygen limitation hypothesis. Despite this, Raby and colleagues (2025) do bring a comprehensive dataset to the field. The results raise questions on whether oxygen supersaturation should be treated as a “rescue factor” in climate risk assessments and ecological models guiding climate-smart conservation.

To advance the field, key questions need to be tackled. First, does oxygen supersaturation in water increase the oxygen transported in blood or hemolymph of all aquatic species? Oxygen limitation is thought to affect water breathers more strongly than air breathers, given lower oxygen concentration and diffusion in water [10]. Yet, this view largely stems from comparisons between aquatic and terrestrial taxa. Greater attention is needed within aquatic species and their diverse breathing modes, and cardiorespiratory systems. For instance, Raby and colleagues (2025) found slightly larger effect sizes in crustaceans than fish, although the role of their distinct cardiorespiratory systems remains unclear. Many aquatic species can also have bimodal respiration, being both water and air-breathers, raising the question of whether they perform better in air than water, or relative to water-breathers alone. Additionally, oxygen limitation may also vary across the life cycle, with early stages being more vulnerable due to underdeveloped organ systems. However, life-stage studies remain scarce. Ultimately, oxygen’s role in contributing to thermal tolerance limits may depend on the medium, life-stage, cardiorespiratory capacity, and oxygen transport pathways (diffusion *versus* pigment-based).

Fundamentally, the central question is whether oxygen limitation represents a universal mechanism shaping thermal tolerance in ectotherms, or whether other mechanisms could be at play, either independently or in combination. A new concept of oxygen-independent thermal tolerance has surfaced for species that maintain cardiorespiratory performance and oxygen supply to tissues at extreme temperatures. Alternative mechanisms proposed to set thermal limits include the temperature-dependent deterioration of electrical excitability, resulting in neural or muscular failure; synaptic dysfunction due to altered membrane fluidity; and mitochondrial dysfunction [11]. The prevalence or concomitant action of such mechanisms across species, populations, and environmental contexts is currently unknown. Either way, fundamental molecular processes related to protein stability, reaction rates, and membrane fluidity are thought to underpin, possibly at different extents, the described physiological-level mechanisms [11].

Future work should explore organisms as highly complex entities, probing mechanisms across levels of biological organization, combining high-throughput (e.g., multi-omics), traditional physiology, and integrative modeling approaches (e.g., network science) [12]. This should help to critically test and understand what unifying or unique mechanisms shape thermal tolerance across ectotherms.

## Abbreviation:

CTmax: Critical Thermal Maximum
